# *FOXP2 *gene and language impairment in schizophrenia: association and epigenetic studies

**DOI:** 10.1186/1471-2350-11-114

**Published:** 2010-07-22

**Authors:** Amparo Tolosa, Julio Sanjuán, Adam M Dagnall, María D Moltó, Neus Herrero, Rosa de Frutos

**Affiliations:** 1 Department of Genetics, Faculty in Biology, University of Valencia, C/Doctor Moliner 50, CP:46100, Burjassot, Valencia, Spain; 2 Psychiatric Unit, Faculty in Medicine, University of Valencia, Avda Blasco Ibáñez 15, CP46010, Valencia, Spain; 3 CIBERSAM, ISCIII, Avda Blasco Ibáñez 15, CP:46010, Valencia, Spain; 4 SANE POWIC, Warnerford Hospital, OX3 7JX, Oxford, UK

## Abstract

**Background:**

Schizophrenia is considered a language related human specific disease. Previous studies have reported evidence of positive selection for schizophrenia-associated genes specific to the human lineage. *FOXP2 *shows two important features as a convincing candidate gene for schizophrenia vulnerability: *FOXP2 *is the first gene related to a language disorder, and it has been subject to positive selection in the human lineage.

**Methods:**

Twenty-seven SNPs of *FOXP2 *were genotyped in a cohort of 293 patients with schizophrenia and 340 controls. We analyzed in particular the association with the poverty of speech and the intensity of auditory hallucinations. Potential expansion of three trinucleotide repeats of *FOXP2 *was also screened in a subsample. Methylation analysis of a CpG island, located in the first exon of the gene, was performed in post-mortem brain samples, as well as qRT-PCR analysis.

**Results:**

A significant association was found between the SNP rs2253478 and the item Poverty of speech of the Manchester scale (p = 0.038 after Bonferroni correction). In patients, we detected higher degree of methylation in the left parahippocampus gyrus than in the right one.

**Conclusions:**

*FOXP2 *might be involved in the language disorder in patients with schizophrenia. Epigenetic factors might be also implicated in the developing of this disorder.

## Background

It is widely accepted that neutral drift and Darwinian positive selection have played an important role in the evolution of human features. During the last few years, research has been focused on human genome-wide scans of adaptative evolving loci to search for specific modern characteristics in this species [1]. Although most of them are related to fitness, it has been reported that some genes under positive selection in the human lineage can also confer vulnerability to some diseases [2-4].

Schizophrenia, which is considered as a disease related to the origin of *Homo sapiens*, could be a by-product of an adaptative process [3,5,6]. Previous reports have indicated a relationship between positively selected genes and schizophrenia. Crespi et al. [3] found signals of positive selection in 28 of 76 schizophrenia candidate genes that had been previously reported as positive results in association studies. Evidence of recent positive selection in the human lineage has also been found in haplotypes of *MAOB *and *GABRB2 *genes, which also confer an increased risk to schizophrenia [2,4]. Furthermore, brain areas that are differentially dysregulated in schizophrenia include the regions most-notably subject to differential evolutionary change along the human lineage [7-9]. In addition, it has recently been suggested that metabolic processes altered in schizophrenia evolved at a higher rate in the human lineage, when compared with the chimpanzee [10].

A selective advantage could affect the achievement of specific human capacities, such as language. In this context, TJ Crow [9,11], postulates that schizophrenia is the price that *Homo sapiens *had to pay for the acquisition of language. Moreover, recent neuroimaging studies report impairment in brain function relevant to language processing in individuals with schizophrenia and in those who are at a genetic risk for this disease [12].

First evidence for a gene involved in language was reported in 2001, when the *FOXP2 *gene was identified by Lai et al. [13]. Identification of the transcriptional targets of FOXP2 revealed that this protein could regulate genes involved in development and function of the brain, genes under positive selection in human lineage and genes associated to schizophrenia [14]. Apart from the polyglutamine tracts, the human protein only differs in three amino acids from its ortholog in mouse, and two of these changes occurred in the human lineage after separation from the common ancestor shared with chimpanzees. Both changes are fixed in human populations, and there is evidence to support they have been under positive selection [15,16].

Association studies between *FOXP2 *polymorphisms and susceptibility to different pathologies of language impairment, such as specific language impairment, dyslexia or autism have not produced robust results [17], but the identification of two coding mutations related to verbal dyspraxia [18]. Nevertheless there are strong evidence of the importance of the gene in development and some aspects of language [19] including the fact that CNTNAP2, a downstream target of FOXP2 has been related also to language disorders [20,21]. In schizophrenia, preliminary association studies have delivered controversial results [22-24]. To the best of our knowledge, no methylation study of *FOXP2 *has previously been done.

We hypothesized that *FOXP2 *could be considered a candidate gene that may confer vulnerability to schizophrenia or to the language related symptoms of this disorder. To test this hypothesis, two different analyses were carried out: 1) an association study between *FOXP2 *polymorphisms and schizophrenia and 2) the study of the methylation status of the *FOXP2 *promoter in different areas of the brain in patients and controls.

## Methods

### Association study participants

For the association study, 293 patients and 340 healthy unrelated controls were analyzed. All patients and controls were Caucasians of Spanish descent. Exclusion criteria included organic brain syndromes, mental retardation, severe drug abuse, or inability to understand simple questions. Participants with previous psychiatric treatment were excluded as controls.

There were no significant differences in sex or age for both groups. All patients met DSM-IV criteria for schizophrenia. The Manchester scale [25], and the psychotic symptom rating scale (PSYRATS) [26], were used respectively, to assess the clinical psychotic symptoms, with particular attention to the Poverty of speech item, and the intensity of auditory hallucinations. The mean Manchester score was 8.79 (SD = 5.56) and mean PSYRATS score was 16.26 (SD = 23.26). This study was approved by the local Ethics Committee. All patients signed the informed consent form.

### Post-mortem human brain samples

For methylation and expression analyses, human brain samples were kindly donated by the London Neurodegenerative Diseases Brain Bank at the Institute of Psychiatry. Grey tissue from both hemispheres of the superior temporal gyrus, parahippocampus gyrus and cingulate gyrus was obtained. For methylation analyses, one sample for each region was analyzed for both patients and controls. For expression analyses, 13 samples from patients (6 from the right hemisphere and 7 from the left hemisphere) and 12 samples from controls (9 from the right and 3 from the left hemisphere) were analyzed.

### Association study

Genomic DNA was extracted from peripheral blood leukocytes by the Puregene kit (Gentra Systems, MN, USA).

A total of 27 polymorphisms were analyzed, 10 of them by polymerase chain reaction-restriction fragment length polymorphism (PCR-RFLP) and 17 by an iPLEX genotyping assay (Sequenom, CA, USA). Details of the primer sequences, PCR conditions and restriction enzymes are described in Additional files 1 and 2.

Three regions were screened for potential trinucleotide expansions: two polyQ tracts of 40 and 10 residues, located respectively in exons 5 and 6 of *FOXP2*, and a CGG-rich region in intron s1 close to the transcription start site. Primers flanking the three regions were designed (see Additional file 1). One primer in each pair was 5'-labeled with 6-FAM or HEX fluorophores. Fluorescent amplicons were electrophoresed with internal lane size standards in an ABI PRISM^® ^3700 DNA Analyzer (Applied Biosystems Inc.) and length of fragments was analyzed with the GeneScan-v3.7 (Applied Biosystems, Inc.).

Statistical and genetic analyses were performed using Haploview v4.1, UNPHASED 3.10, and SSPS v13 software. Bonferroni correction was used for multiple tests. For the haplotype association study, four marker sliding-windows were used, with the exception of a five marker haplotype, for which association had been detected in a previous study [24].

### Methylation analysis

DNA from brain samples was extracted using a Nucleon^® ^Genomic DNA Extraction Kit (Tepnel Life Sciences). DNA from leukocyte samples was extracted using the Puregene kit (Gentra Systems).

DNA was fragmented with *Eco*RI (New England Biolabs) prior to overnight digestion with Proteinase K (Sigma Aldrich). DNA was cleaned, purified and concentrated using a Qiaex II kit (Qiagen). The processed DNA samples were treated with either the CpGenome™ DNA Modification Kit (Chemicon^® ^International) or the EpiTect Bisulfite Kit (Qiagen) in accordance with the supplier's guidelines.

DNA was amplified with specific primers for bisulphite-converted DNA (see Additional file 1). PCR fragments were cloned into the PCR 2.1 vector using the TOPO cloning kit (Invitrogen), or pGEM-T^® ^vector using the pGEM-T^® ^Easy Vector System (Promega), and sequenced with T7 and SP6 universal primers.

### Expression analysis

Total RNA was extracted with the RNeasy Lipid Tissue Mini Kit (Qiagen). Reverse transcription of 1 μg of RNA was performed using SuperScriptTM III Reverse Transcriptase (Invitrogen) and random primer hexanucleotides (Promega).

Quantitative RT-PCR was performed in triplicate for each sample on an iCycler iQ Real Time PCR System (Qiagen) with Power SYBR^® ^Green PCR Master Mix (Applied Biosystems) using a standard protocol. Specific cDNA primers for *FOXP2 *and *RPII*, used as a control gene, were designed. Sequences and PCR conditions are shown in Additional file 1. The comparative CT method (ΔΔCT) was used to measure the relative gene expression.

## Results

### Association study

**Figure 1:**
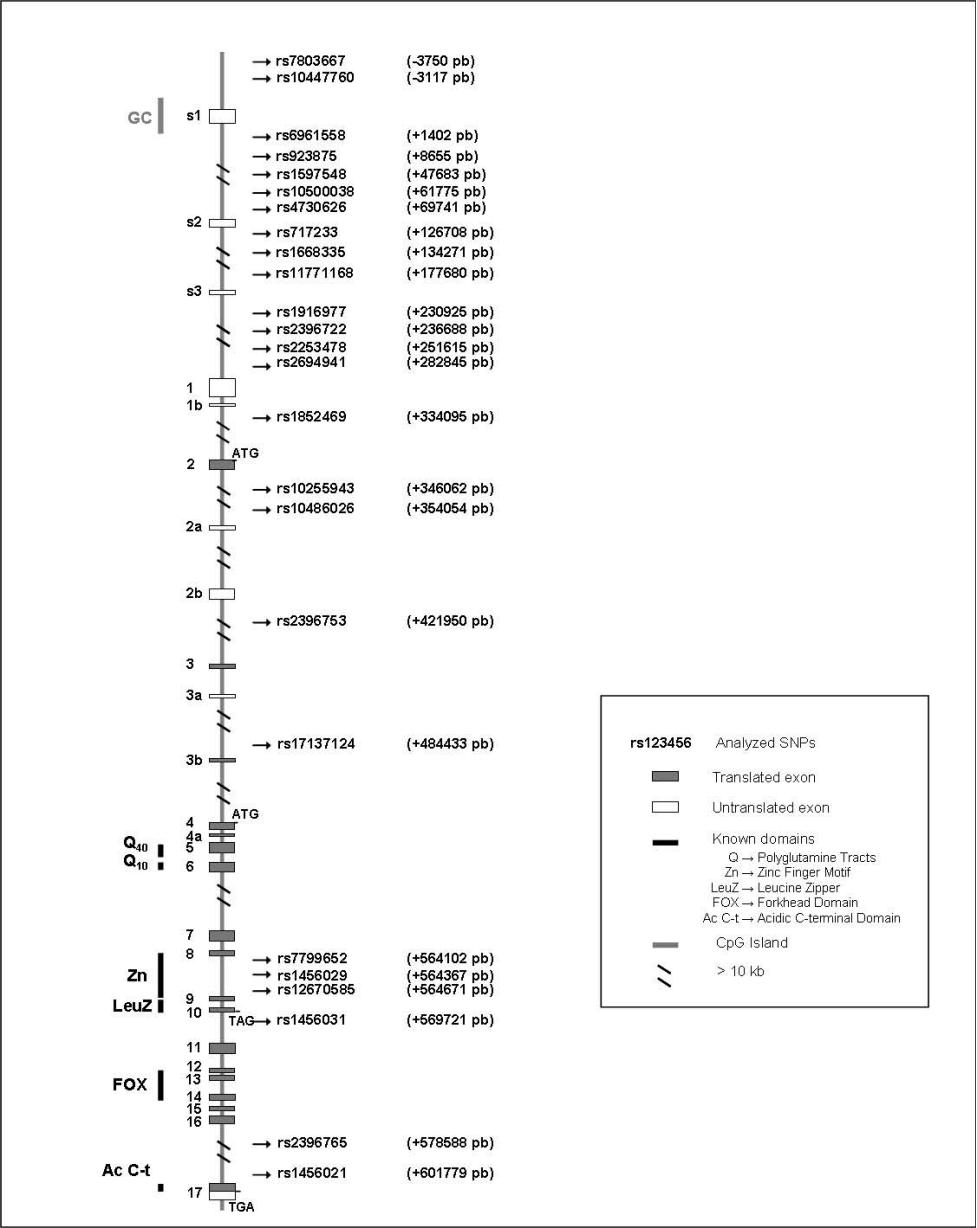
**Structure of *FOXP2 *gene**. Hash marks indicate introns longer than 50 kb. Arrows indicate positions of all single nucleotide polymorphisms (SNPs) analyzed in this study: arrows indicate SNPs polymorphic in our sample. Distances of SNPs to +1 site (5' end of s1 exon) are shown in brackets.

Twenty-four intronic SNPs and three SNPs within the 5' untranslated region of *FOXP2 *were selected for this study. The SNPs rs13308496 and rs10254225 were monomorphic in our sample. In Figure 1, we show the location of the selected SNPs that were polymorphic in our sample. All of the SNPs were at Hardy-Weinberg equilibrium in both patients and controls, except rs717233, which deviated from HWE in controls (see Additional file 3) and was removed in further analyses. Allelic and genotypic frequencies of the twenty-four SNPs in both patients and controls are shown in Additional file 4. Minor allele frequencies for the SNPs ranged between 0.02 and 0.49.

First, we conducted the single SNP association analysis. When all patients were included, we observed a significant association for the SNP rs10447760 at allelic frequencies, although this association did not remain after applying the conservative Bonferroni correction. When comparing patients with auditory hallucinations versus controls (see Additional file 5), significant associations were found for SNP rs2396753 and SNP rs17137124. However, after Bonferroni correction, the significant associations were also lost.

Finally, when patients with auditory hallucinations were compared with patients without hallucinations (see Additional file 6), a significant association was found for SNP rs2253478 and SNP rs1456031 in genotypic frequencies and for SNP rs2396753 in both, genotypic and allelic frequencies. However, once again, after Bonferroni correction, the significance disappeared in all cases.

Next, we carried out the haplotypic association analysis. Tests of four marker haplotypes did not provide evidence of significant associations with schizophrenia or auditory hallucinations. However, when we took into account the five marker haplotype which was found to be significant in a previous study [24], positive results were detected for the same combination of alleles: rs7803667T/rs10447760C/rs923875A/rs2396722C/rs2396753A (χ2 = 6.479; p = 0.0109). Interestingly, this combination of alleles was found more frequent in controls than in schizophrenic patients with auditory hallucinations.

Linear regression was performed to evaluate the association between the SNPs and the items of the PSYRATS and Manchester scales. After Bonferroni correction was applied, only association between SNP rs2253478 and the Poverty of speech was maintained (p corrected = 0.038).

With regard to the analyses of potential expansions of trinucleotide tracts of *FOXP2*, no variation was found in any of these regions in our sample. Only a single deletion of three trinucleotides at the CGG-rich region in intron s1 was identified in heterozygosis in a patient with schizophrenia.

### Methylation analyses

**Figure 2:**
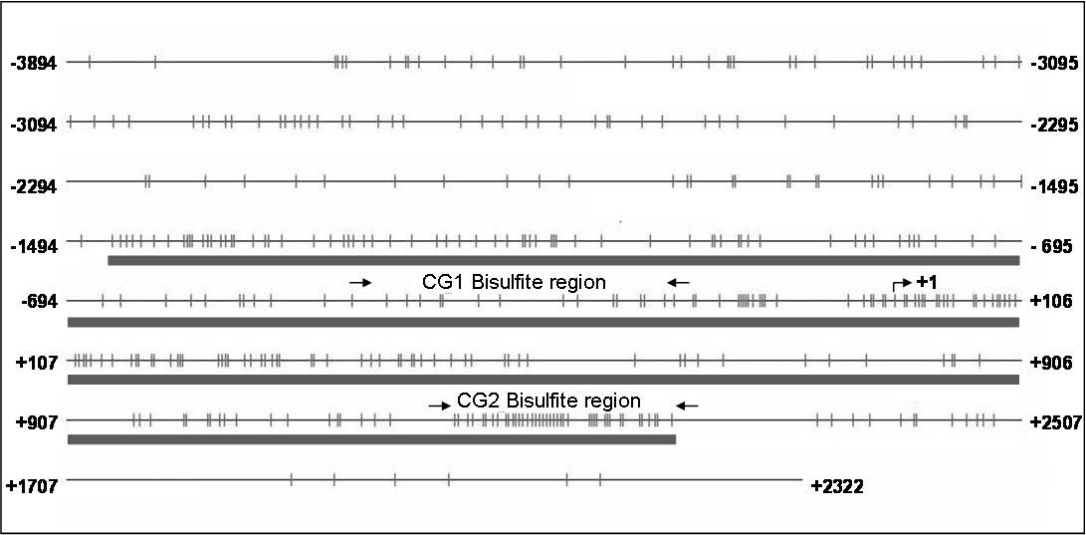
**CpG dinucleotides in the context of the CpG island located in transcription start site**. The thin black line corresponds to the sequence of DNA. Below this, a single black bar corresponds to the predicted CpG island location. Distances in base pairs to +1 site are included, as well as an arrow indicating the start site of exon s1. Primers for both regions analyzed, CG1 and CG2 are indicated with arrows.

*FOXP2 *has four independent transcriptional start sites that would be termed exon s1, exon 1, exon 1b and exon 2 using the conventional designations of its initiation exon. In this work, we have focused on the study of region including exon s1, which is considered a basal transcription start site. Figure 2 shows a diagram of the region studied.

**Figure 3:**
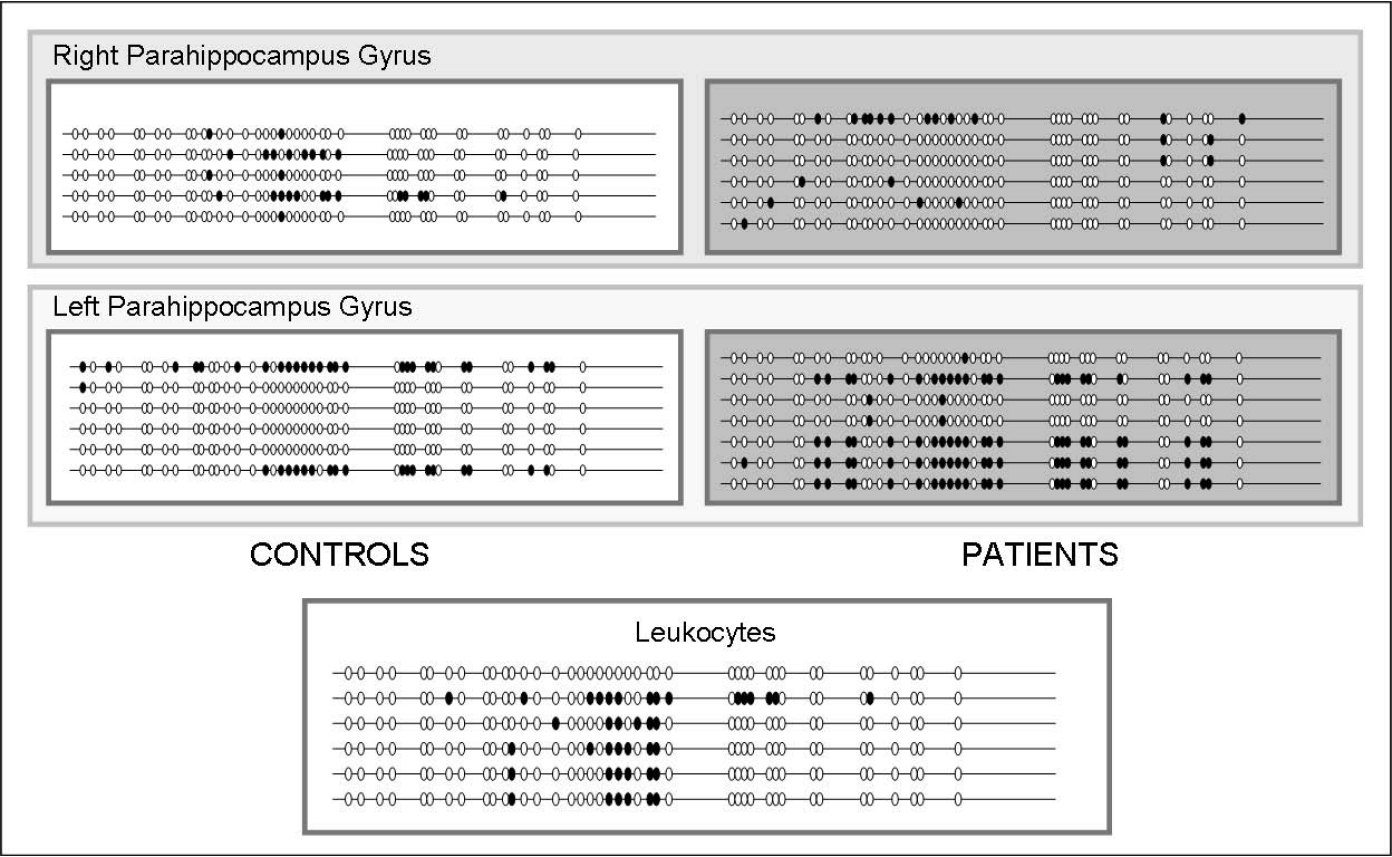
**Bisulfite results for parahippocampus gyrus**. Methylation data are represented as filled circles (methylated CpG) and empty circles (unmethylated CpG) for each bacterial clone obtained. Each row of circles represents the methylation pattern based on the sequence of one cloned PCR product.

For the region named CG1 bisulphite region, no differences were found between patients and controls or between brain areas. This region, located upstream of exon s1 was characterized by a general absence of methylation in all the samples analyzed. In the case of CG2 bisulphite region, located downstream of exon s1, a higher degree of methylation was found with respect to CG1. In addition, subtle differences were found for the parahippocampus gyrus. Within this brain area, the methylation degree is higher than in the other areas analyzed in both patients and controls. When right and left parahippocampus gyrus regions were compared, differences in the degree of methylation between patients and controls were identified (Figure 3). In patients, the degree of methylation is higher in the left hemisphere of the parahippocampus gyrus than in the right one, whereas in controls, methylation is concentrated more in the right hemisphere of the parahippocampus gyrus region. Interestingly, most of the clones that were analyzed showed similar methylation patterns (Figure 3). A high degree of methylation in the CG2 region was also found in leukocyte samples of controls. In this case, the observed methylation pattern is different from the one obtained from brain samples of controls.

**Figure 4:**
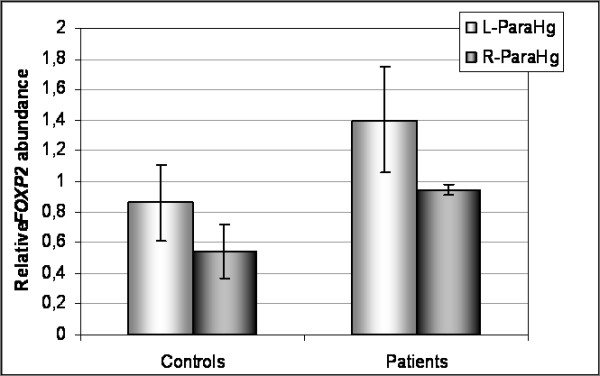
**Levels of *FOXP2 *expression in parahippocampus gyrus brain area**.

Generally, a high degree of methylation on the promoter region of a gene is correlated with lower RNA expression levels. Therefore, differences in the degree of methylation could result in differential expression of the gene. When comparing the relative expression level of *FOXP2 *in parahippocampus gyrus between patients and controls, higher levels of *FOXP2 *mRNA expression were obtained in the right hemisphere in patients (Figure 4). This correlates inversely with methylation results, which show a greater degree of methylation in controls than in patients. However, when expression levels from the left and right hemispheres were compared in patients, no correlation was found, since expression is higher in left parahippocampus than in right, as well as the level of methylation. These findings do not support the idea that a high degree of methylation leads to decreased expression of the gene.

## Discussion

In this study we investigated the role of *FOXP2*, a positively selected gene, in schizophrenia vulnerability. A SNP association study, with particular attention to language related symptoms as auditory hallucinations and poverty of speech, and a study of DNA methylation and expression of this gene were carried out.

The most important finding of this study is the significant association showed between the rs2253478 SNP and the item of Poverty of speech of the Manchester scale (p = 0.038 after Bonferroni correction). This polymorphism is located in intron s3, not close to any of the promoter regions. There is no information that it could be an enhancer of splicing element. Its potential functionality has not been yet investigated, and then it is difficult to determine the biological significance of this association. Alternatively it could be in linkage disequilibrium with another polymorphism being the causative factor. In any case, our results relate the *FOXP2 *gene to one of the characteristic symptoms of schizophrenia, deficits in the language domain [27-29].

On the other hand, the haplotypic analysis confirmed our previous results that the rs7803667T/rs10447760C/rs923875A/rs2396722C/rs2396753A haplotype could be a protective one with respect to auditory hallucinations [24].

It has been suggested that specific language-related circuits are affected in patients with schizophrenia [12]. Therefore, it is reasonable to look for risk alleles to schizophrenia vulnerability in *FOXP2*, a gene for which an implication in the development of language is well accepted [13,30]. Nevertheless, schizophrenia is a biological entity not well defined, indicative of a phenotype too much complex for genetic analysis, which partially explains the difficulty to find the causative genetic factors. At this point, the study of language variables in order to find risk alleles in schizophrenia becomes a good alternative with respect to endophenotype approaches. Our results support this hypothesis, since significant results were found when we related language impairment in schizophrenic patients to *FOXP2 *polymorphisms.

In this work, we also analyzed whether the polyQ stretches at exons 5 and 6 of *FOXP2 *are polymorphic and if so, determine its potential association with schizophrenia vulnerability. Expansions in the number of trinucleotides repeats are frequently associated with neurodegenerative diseases [31]. However, no variation in the number of glutamines was found in our sample. This high stability is concordant with previous studies in controls, individuals with progressive movement disorders, and schizophrenic patients [32,33]. The role of the polyQ tracts in the *FOXP2 *gene is unknown. In fact, most of the members of the FOX family lack this domain. Nevertheless, the high invariability of these sequences suggests that they could be under functional constraints.

In addition to schizophrenia vulnerability due to variations in the DNA sequence, epigenetic factors regulating gene expression have also been suggested as a potential etiological mechanism in psychosis [34,35]. Epigenetic regulation has been increasingly associated with psychiatric disorders, with examples in depression and addiction [36,37].

In our study of DNA methylation of *FOXP2 *exon s1 region, we found a higher degree of methylation in the left hemisphere of the parahippocampus gyrus region in patients than in controls. From these results, we would have expected lower gene expression of *FOXP2 *due to repression by methylation. However, no differences were found in *FOXP2 *expression between controls and patients. This discrepancy could be explained by the fact that only a stretch of the CpG island, located in exon s1, was analyzed for methylation. The promoter region of the *FOXP2 *gene has not been well defined, and regulation of the gene is more complex than was initially thought (non published personal data, [38]). The finding that expression data show a trend of more expression in patients than in controls would indicate that a decrease of neural processes controlled by the protein FOXP2, a repressor of transcription, is produced in patients. Hippocampal and parahippocampal volume reduction is one of the most consistent findings in schizophrenia [39]. Moreover, in a meta-analysis of brain volumes in relatives of patients with schizophrenia, hippocampal reduction was the largest difference between relatives and healthy controls [40]. These findings suggest hippocampal volume as a potential end of phenotype for genetic studies in schizophrenia.

Our study has some limitations. First, the language skills evaluated in this work include only two items of the Manchester scale. Since the strongest result is related to one of these items, we would recommend a systematic exploration of language variables in schizophrenic patients. Therefore, it would be valuable to explore different aspects of language in future studies. Second, we have used a small sample in the methylation and expression analyses so further studies with a larger sample would be necessary in order to confirm our preliminary results. Finally, other variables which could affect methylation, such as medication or age, should be considered. In spite of these limitations, this study suggests the use of the language related disorder as alternative phenotypes in schizophrenia for genetic studies. On the other hand, although the results are not conclusive, this is the first epigenetic study of *FOXP2 *in schizophrenia, opening a new way in which this gene could be related to this disorder.

## Conclusions

Our results do not support the involvement of *FOXP2 *in the vulnerability to schizophrenia as a global syndrome. Nevertheless, this gene might be implicated in schizophrenia through its role in language impairment. Epigenetic mechanisms affecting the expression of *FOXP2 *might contribute to the development of schizophrenia and related neurodevelopmental disorders.

## Competing interests

The authors declare that they have no competing interests.

## Authors' contributions

AT participated in the experimental procedure, analysis of results and the draft of the manuscript. AMD participated in the experimental procedure and the draft of the manuscript. MDM, and JS participated in the conception and the design of the study. RF conceived of the study, and participated in its design and coordination and helped to draft the manuscript. All authors contributed to and have approved the final manuscript.

## Pre-publication history

The pre-publication history for this paper can be accessed here:

http://www.biomedcentral.com/1471-2350/11/114/prepub

## Supplementary Material

Additional file 1 **Sequences of primers used in the association study, analysis of potential expansions of trinucleotides, methylation analyses and quantitative PCR**. For SNP rs6961558, one of the primers was modified in order to create a restriction enzyme target depending on the allele in the sequence. Modified nucleotide is shown in grey. Click here for file

Additional file 2 **RFLPs conditions**. Click here for file

Additional file 3 **Results for Hardy-Weinberg equilibrium test in patients and controls**. Click here for file

Additional file 4 **Genotype and allele frequencies of the analyzed SNPs in patients and controls**. ^a ^tests in which expected values for more than one class are lower than 5. * it corresponds to corrected p value (Bonferroni correction). Click here for file

Additional file 5 **Genotype and allele frequencies of the analyzed SNPs in patients with auditory hallucinations (AH) and controls**. ^a ^tests in which expected values for more than one class are lower than 5. ^b ^tests in which due to lack of some classes, it was used a table 2 × 2 instead a 3 × 2. * it corresponds to corrected p value (Bonferroni correction). Click here for file

Additional file 6 **Genotype and allele frequencies of the analyzed SNPs in patients with auditory hallucinations (AH) and patients without AH**. ^a ^tests in which expected values for more than one class are lower than 5. ^b ^tests in which expected values for one class are lower than 2. ^c ^tests in which due to lack of some classes, it was used a table 2 × 2 instead a 3 × 2. * it corresponds to corrected p value (Bonferroni correction). Click here for file
