# Supplementary material for: FOXP2 gene and language impairment in schizophrenia: association and epigenetic studies
Source: BMC Med Genet. 2010 Jul 22;11:114. doi: 10.1186/1471-2350-11-114 (PMC2918571; doi:10.1186/1471-2350-11-114)
Supplement: Additional file 1 — Sequences of primers used in the association study, analysis of potential expansions of trinucleotides, methylation analyses and quantitative PCR. For SNP rs6961558, one of the primers was modified in order to create a restriction enzyme target depending on the allele in the sequence. Modified nucleotide is shown in grey. [file 1471-2350-11-114-S1.PDF]

Polimorphism	Primers (5'-3')	PCR lenght	Annealing temperature
	RFLPs		
rs7803667	F: ACCCTTGATTTGACTTCGGTG<br>R: TCGAGACACTGCTCTAGTACG	641	61ºC
rs10447760	F: CTATTCGATCGCTGTTTGCC<br>R: GTGGCACGTAGTTTGTTGATG	248	56ºC
rs6961558	F: GAGCGCAGACACCTTTCGGTG<br>R: CACTGAGGTCGGGTGTCACGG	263	56ºC
rs923875	F: CTTGGGAAACTGAAGCCAG<br>R: ACTCACCCAATATCATGCAATAG	625	61ºC
rs1597548	F: CAATTCACCAACAGTTGTACC<br>R: GATGGCTACATCTGCTTTGAC	410	59ºC
rs2396722	F: GTCATCAATGTCACAGAGAACTTG<br>R: AGACTGTACTTGTTCCTGGGAG	830	62ºC
rs1852469	F: GGCTACAGTTTACAAGACACCAGG<br>R: GTCCAGCCTTTGGGAATTTGAC	299	60ºC
rs2396753	F: TGGCTATGAAATAACAAGCACAAC<br>R: CCCAGTTATTGGCTCACTCTACC	251	60ºC
rs17137124	F: GGTTCCTACAGCAGTATCATGG<br>R: TTATCTGCACCAATGGAAGG	470	57ºC
rs1456031	F: CAAAGTTATCAAGGCTGCGAGTC<br>R: CATCTTTTTCAATGCAAACCACTCA	241	60ºC
	Trinucleotide repeats primers		
40 polyglutamines tract	6-FAM-F: GCAAGAGCAGTTACATCTTC<br>R: ATGAGATAACCGGATCCTAC	213	58ºC
10 polyglutamine tract	HEX-F: CTCTAGACCTTGCTCCATAC<br>R: TGTTGCATCTGGAGAAGCTG	320	60.5ºC
CGG tract	6-FAM-F: GAGCGCAGACACCTTTCGGTG<br>R: TGCGGAGCGTCCCAAGCGGTG	517	61ºC
	Bisulfite primers		
CG1 Bisulfite region	F: GCGGGTTGTTTATATAGTAGGTGGATT<br>R: CAAAACTACCTCTCCTCGAAAAAA	289	61ºC
CG2 Bisulfite region	F: GTTATTTGGAAGTTTATAGTGGTT<br>R: TAACTTTTCCTCCCTACTCTAAAA	274	59ºC
	Quantitative PCR primers		
FOXP2-RT	F: GGTGCAACAGTTAGAAATACAG<br>R: ACCAGATTTAGAGGTTTGGGA	116	60ºC
RPII-RT	F: GTGCGGCTGCTTCCATAA<br>R: GCACCACGTCCAATGACAT	228	60ºC
