# Supplementary material for: FOXP2 gene and language impairment in schizophrenia: association and epigenetic studies
Source: BMC Med Genet. 2010 Jul 22;11:114. doi: 10.1186/1471-2350-11-114 (PMC2918571; doi:10.1186/1471-2350-11-114)
Supplement: Additional file 2 — RFLPs conditions. [file 1471-2350-11-114-S2.PDF]

SNP	Restriction enzyme	Digestion temperature	PCR lenght (bp)	Restriction lenght fragments (bp)[a]	% agarose gel
rs7803667	*Tas*I	65ºC	641	Allele A: 42+156+18+53+37+201 +105+6 +23<br>Allele T: 42+71+85+18+53+37 +201+105 +6+23	12% Polyacrylamide gel
rs10447760	*Hpa*II	37ºC	248	Allele T: 50+198<br>Allele C: 50+62+136	2
rs6961558	*Taa*I	65ºC	263	Allele G: 62+201<br>Allele A: 62+180+21	3
rs923875	*Alw*44I	37ºC	625	Allele A: 625<br>Allele C:237+388	2
rs1597548	*Acc*I	37ºC	410	Allele C: 410<br>Allele A: 179+231	2
rs2396722	*Vsp*I	37ºC	830	Allele C: 315+515<br>Allele T: 315+443+72	2.5
rs1852469	*Tru*1I	65ºC	299	Allele T: 84+215<br>Allele A: 84+124+91	2
rs2396753	*Eam*1104I	37ºC	251	Allele C: 251<br>Allele A: 125+126	2
rs17137124	*Bsp*TI	37ºC	470	Allele C: 470<br>Allele T: 240+230	2
rs1456031	*Cac*8I	37ºC	241	Allele C: 241<br>Allele T:149+92	2
