# Supplementary material for: FOXP2 gene and language impairment in schizophrenia: association and epigenetic studies
Source: BMC Med Genet. 2010 Jul 22;11:114. doi: 10.1186/1471-2350-11-114 (PMC2918571; doi:10.1186/1471-2350-11-114)
Supplement: Additional file 3 — Results for Hardy-Weinberg equilibrium test in patients and controls. [file 1471-2350-11-114-S3.PDF]

SNPs	P values for HWE test		SNPs	P values for HWE test	
	Controls	Patients		Controls	Patients
rs7803667	1.0	0.3744	rs2694941	0.215	0.6228
rs10447760	0.7394	0.2217	rs1852469	0.3018	0.7478
rs6961558	1.0	1.0	rs10255943	1.0	0.7105
rs923875	1.0	0.2088	rs10486026	0.3348	0.9273
rs1597548	1.0	0.9671	rs2396753	0.0638	0.9787
rs10500038	0.3499	1.0	rs17137124	0.0596	0.4863
rs4730626	0.204	0.8688	rs7799652	0.7255	0.5769
rs717233	0.0391	0.4304	rs1456029	0.3627	0.9972
rs1668335	0.7392	0.3942	rs12670585	0.7125	0.5963
rs11771168	0.9577	0.8903	rs1456031	0.3718	1.0
rs1916977	0.1774	0.7229	rs2396765	0.7525	0.5808
rs2396722	0.6377	1.0	rs1456021	0.6308	0.7897
rs2253478	0.7714	0.5998			
