# Supplementary material for: FOXP2 gene and language impairment in schizophrenia: association and epigenetic studies
Source: BMC Med Genet. 2010 Jul 22;11:114. doi: 10.1186/1471-2350-11-114 (PMC2918571; doi:10.1186/1471-2350-11-114)
Supplement: Additional file 4 — Genotype and allele frequencies of the analyzed SNPs in patients and controls. a tests in which expected values for more than one class are lower than 5. * it corresponds to corrected p value (Bonferroni correction). [file 1471-2350-11-114-S4.PDF]

SNP		Genotype frequencies			χ^2	P	Allelic frequencies		χ^2	P[b]
**rs7803667**		**TT**	**TA**	**AA**			**T**	**A**		
Controls	340	0.5	0.42	0.09	**3.145**	**0.208**	0.71	0.29	**2.587**	**0.108**
Patients	293	0.43	0.47	0.1			0.66	0.34		
**rs10447760**		**CC**	**CT**	**TT**			**C**	**T**		
Controls	340	0.57	0.37	0.06	**5.596**	**0.061**	0.75	0.25	**3.993**	**0.046 1.00**
Patients	293	0.47	0.45	0.07			0.70	0.30		
**rs6961558**		**GG**	**GA**	**AA**			**G**	**A**		
Controls	339	0.95	0.05	0	**0.666**	**0.431**	0.98	0.02	**0.652**	**0.419**
Patients	292	0.97	0.03	0			0.98	0.02		
**rs923875**		**AA**	**AC**	**CC**			**A**	**C**		
Controls	340	0.37	0.48	0.15	**1.597**	**0.455**	0.61	0.39	**0.831**	**0.362**
Patients	293	0.32	0.53	0.15			0.58	0.42		
**rs1597548**		**CC**	**CG**	**GG**			**C**	**G**		
Controls	340	0.88	0.11	0.002	**1.122**	**0.840**	0.94	0.06	**0.492**	**0.483**
Patients	293	0.9	0.1	0			0.95	0.05		
**rs10500038**		**CC**	**CT**	**TT**			**G**	**A**		
Controls	319	0.67	0.31	0.02	**1.371**	**0.510**	0.82	0.18	**0.7**	**0.403**
Patients	265	0.65	0.32	0.04			0.80	0.20		
**rs4730626**		**GG**	**GA**	**AA**			**G**	**A**		
Controls	315	0.64	0.3	0.06	**2.124**	**0.349**	0.79	0.21	**1.871**	**0.171**
Patients	262	0.68	0.29	0.03			0.82	0.18		
**rs1668335**		**GG**	**GA**	**AA**			**G**	**A**		
Controls	315	0.52	0.4	0.09	**0.185**	**0.913**	0.72	0.28	**0.011**	**0.917**
Patients	263	0.53	0.38	0.09			**0.72**	**0.28**		
**rs11771168**		**CC**	**CT**	**TT**			**C**	**T**		
Controls	319	0.6	0.35	0.05	**0.234**	**0.897**	0.78	0.22	**0.095**	**0.789**
Patients	265	0.62	0.33	0.05			0.78	0.22		
**rs1916977**		**AA**	**AG**	**GG**			**A**	**G**		
Controls	314	0.58	0.34	0.08	**0.400**	**0.824**	0.75	0.25	**0.019**	**0.892**
Patients	263	0.58	0.36	0.06			0.76	0.24		
**rs2396722**		**TT**	**TC**	**CC**			**T**	**C**		
Controls	340	0.42	0.44	0.14	**0.375**	**0.838**	0.64	0.36	**0.032**	**0.857**
Patients	293	0.41	0.46	0.13			0.64	0.36		
**rs2253478**		**GG**	**GA**	**AA**			**G**	**A**		
Controls	340	0.4	0.44	0.16	**1.900**	**0.402**	0.62	0.38	**0.003**	**0.954**
Patients	293	0.38	0.49	0.13			0.62	0.38		
**rs2694941**		**TT**	**TA**	**AA**			**T**	**A**		
Controls	315	0.35	0.46	0.19	**0.198**	**0.909**	0.58	0.42	**0.001**	**0.979**
Patients	261	0.34	0.48	0.18			0.58	0.42		
**rs1852469**		**AA**	**AT**	**TT**			**A**	**T**		
Controls	340	0.94	0.06	0	**1.863**[a]	**0.388**	0.97	0.03	**1.247**	**0.264**
Patients	293	0.92	0.08	0.003			0.96	0.04		
**rs10255943**		**GG**	**GA**	**AA**			**G**	**A**		
Controls	314	0.49	0.4	0.11	**0.837**	**0.672**	0.69	0.31	**0.001**	**0.973**
Patients	263	0.48	0.43	0.09			0.69	0.31		
**rs10486026**		**TT**	**TC**	**CC**			**T**	**C**		
Controls	315	0.62	0.31	0.07	**2.385**	**0.305**	0.78	0.22	**0.275**	**0.600**
Patients	263	0.62	0.34	0.04			0.79	0.21		
**rs2396753**		**AA**	**AC**	**CC**			**A**	**C**		
Controls	340	0.29	0.55	0.16	**4.860**	**0.089**	0.56	0.44	**3.759**	**0.053**
Patients	293	0.25	0.52	0.23			0.51	0.49		
**rs17137124**		**TT**	**TC**	**CC**			**T**	**C**		
Controls	340	0.25	0.49	0.26	**1.915**	**0.383**	0.49	0.51	**1.825**	**0.177**
Patients	293	0.29	0.47	0.23			0.53	0.47		
**rs7799652**		**TT**	**TG**	**GG**			**T**	**G**		
Controls	319	0.27	0.53	0.2	**2.137**	**0.346**	0.54	0.46	**1.488**	**0.223**
Patients	265	0.33	0.49	0.18			0.57	0.43		
**rs1456029**		**AA**	**AG**	**GG**			**A**	**G**		
Controls	319	0.6	0.34	0.06	**1.685**	**0.427**	0.77	0.23	**1.728**	**0.189**
Patients	265	0.55	0.37	0.08			0.74	0.26		
**rs12670585**		CC	CT	TT			C	T		
Controls	315	0.5	0.4	0.1	**1.025**	**0.608**	0.70	0.30	**0.573**	**0.449**
Patients	262	0.51	0.41	0.08			0.72	0.28		
**rs1456031**		**TT**	**TC**	**CC**			**T**	**C**		
Controls	340	0.29	.49	0.23	**2.387**	**0.308**	0.53	0.47	**2.116**	**0.146**
Patients	293	0.34	0.45	0.2			0.57	0.43		
**rs2396765**		**TT**	**TC**	**CC**			**T**	**C**		
Controls	317	0.35	0.47	0.18	**2.059**	**0.356**	0.59	0.41	**1.598**	**0.206**
Patients	263	0.38	0.48	0.14			0.62	0.38		
**rs1456021**		**TT**	**TG**	**GG**			**T**	**G**		
Controls	334	0.35	0.47	0.18	**3.676**	**0.157**	0.58	0.42	**3.085**	**0.079**
Patients	278	0.39	0.48	0.13			0.63	0.37		
