# Supplementary material for: FOXP2 gene and language impairment in schizophrenia: association and epigenetic studies
Source: BMC Med Genet. 2010 Jul 22;11:114. doi: 10.1186/1471-2350-11-114 (PMC2918571; doi:10.1186/1471-2350-11-114)
Supplement: Additional file 5 — Genotype and allele frequencies of the analyzed SNPs in patients with auditory hallucinations (AH) and controls. a tests in which expected values for more than one class are lower than 5. b tests in which due to lack of some classes, it was used a table 2 × 2 instead a 3 × 2. * it corresponds to corrected p value (Bonferroni correction). [file 1471-2350-11-114-S5.PDF]

SNP		Genotype frequencies			χ^2	P	Allelic frequencies		χ^2	P *
**rs7803667**		**TT**	**TA**	**AA**			**T**	**A**		
Controls	340	0.5	0.42	0.09	**2.240**	**0.328**	0.71	0.29	**2.047**	**0.153**
Patients with AH	215	0.43	0.47	0.1			0.67	0.33		
**rs10447760**		**CC**	**CT**	**TT**			**C**	**T**		
Controls	340	0.57	0.37	0.06	**3.842**	**0.148**	0.75	0.25	**2.673**	**0.102**
Patients with AH	215	0.48	0.45	0.07			0.71	0.29		
**rs6961558**		**GG**	**GA**	**AA**			**G**	**A**		
Controls	339	0.95	0.05	0	**0.317**[b]	**0.369**	0.98	0.02	**0.310**	**0.578**
Patients with AH	215	0.96	0.04	0			0.98	0.02		
**rs923875**		**AA**	**AC**	**CC**			**A**	**C**		
Controls	340	0.37	0.48	0.15	**0.465**	**0.784**	0.61	0.39	**0.275**	**0.600**
Patients with AH	215	0.34	0.51	0.15			0.59	0.41		
**rs1597548**		**CC**	**CG**	**GG**			**C**	**G**		
Controls	340	0.88	0.11	0.002	**0.094**[a]	**0.956**	0.94	0.06	**0.0001**	**0.991**
Patients with AH	215	0.88	0.12	0			0.94	0.06		
**rs10500038**		**CC**	**CT**	**TT**			**G**	**A**		
Controls	319	0.67	0.31	0.02	**3.258**	**0.206**	0.82	0.18	**1.729**	**0.189**
Patients with AH	200	0.63	0.32	0.05			0.79	0.21		
**rs4730626**		**GG**	**GA**	**AA**			**G**	**A**		
Controls	315	0.64	0.3	0.06	**1.492**	**0.494**	0.79	0.21	**1.586**	**0.208**
Patients with AH	200	0.68	0.28	0.04			0.82	0.18		
**rs1668335**		**GG**	**GA**	**AA**			**G**	**A**		
Controls	315	0.52	0.4	0.09	**0.285**	**0.867**	0.72	0.28	**0.003**	**0.955**
Patients with AH	200	0.51	0.42	0.08			**0.72**	**0.28**		
**rs11771168**		**CC**	**CT**	**TT**			**C**	**T**		
Controls	319	0.6	0.35	0.05	**0.415**	**0.822**	0.78	0.22	**0.329**	**0.566**
Patients with AH	200	0.63	0.32	0.04			**0.79**	**0.21**		
**rs1916977**		**AA**	**AG**	**GG**			**A**	**G**		
Controls	314	0.58	0.34	0.08	**0.603**	**0.740**	0.75	0.25	**0.062**	**0.804**
Patients with AH	200	0.58	0.36	0.06			0.76	0.24		
**rs2396722**		**TT**	**TC**	**CC**			**T**	**C**		
Controls	340	0.42	0.44	0.14	**0.094**	**0.956**	0.64	0.36	**0.063**	**0.802**
Patients with AH	215	0.42	0.44	0.13			0.64	0.36		
**rs2253478**		**GG**	**GA**	**AA**			**G**	**A**		
Controls	317	0.4	0.44	0.16	**0.215**	**0.904**	0.62	0.38	**0.143**	**0.705**
Patients with AH	199	0.41	0.44	0.15			0.63	0.37		
**rs2694941**		**TT**	**TA**	**AA**			**T**	**A**		
Controls	315	0.35	0.46	0.19	**0.172**	**0.915**	0.58	0.42	**0.049**	**0.824**
Patients with AH	198	0.33	0.47	0.19			0.57	0.43		
**rs1852469**		**AA**	**AT**	**TT**			**A**	**T**		
Controls	340	0.94	0.06	0	**2.226**[a]	**0.329**	0.97	0.03	**1.342**	**0.246**
Patients with AH	215	0.92	0.08	0.005			0.96	0.04		
**rs10255943**		**GG**	**GA**	**AA**			**G**	**A**		
Controls	314	0.49	0.4	0.11	**0.650**	**0.730**	0.69	0.31	**0.015**	**0.904**
Patients with AH	200	0.47	0.44	0.1			0.69	0.31		
**rs10486026**		**TT**	**TC**	**CC**			**T**	**C**		
Controls	315	0.62	0.31	0.07	**2.930**	**0.234**	0.78	0.22	**0.657**	**0.418**
Patients with AH	200	0.63	0.34	0.04			0.79	0.21		
**rs2396753**		**AA**	**AC**	**CC**			**A**	**C**		
Controls	340	0.29	0.55	0.16	**9.269**	**0.010**<br>**0.24**	0.56	0.44	**8.183**	**0.004**<br>**0.096**
Patients with AH	215	0.21	0.53	0.26			0.48	0.52		
**rs17137124**		**TT**	**TC**	**CC**			**T**	**C**		
Controls	340	0.25	0.49	0.26	**4.137**	**0.124**	0.49	0.51	**4.209**	**0.040**<br>**0.96**
Patients with AH	215	0.32	0.48	0.2			0.56	0.44		
**rs7799652**		**TT**	**TG**	**GG**			**T**	**G**		
Controls	319	0.27	0.53	0.2	**2.530**	**0.293**	0.54	0.46	**2.385**	**0.123**
Patients with AH	200	0.33	0.51	0.16			0.58	0.42		
**rs1456029**		**AA**	**AG**	**GG**			**A**	**G**		
Controls	319	0.6	0.34	0.06	**2.247**	**0.325**	0.77	0.23	**2.164**	**0.141**
Patients with AH	200	0.55	0.36	0.08			0.73	0.27		
**rs12670585**		CC	CT	TT			C	T		
Controls	315	0.5	0.4	0.1	**1.244**	**0.553**	0.70	0.30	**0.639**	**0.424**
Patients with AH	199	0.51	0.41	0.08			0.72	0.28		
**rs1456031**		**TT**	**TC**	**CC**			**T**	**C**		
Controls	340	0.29	0.49	0.23	**3.842**	**0.148**	0.53	0.47	**3.242**	**0.072**
Patients with AH	215	0.34	0.49	0.17			0.59	0.41		
**rs2396765**		**TT**	**TC**	**CC**			**T**	**C**		
Controls	317	0.35	0.47	0.18	**3.240**	**0.203**	0.59	0.41	**1.716**	**0.192**
Patients with AH	198	0.37	0.51	0.12			0.63	0.37		
**rs1456021**		**TT**	**TG**	**GG**			**T**	**G**		
Controls	334	0.35	0.47	0.18	**4.473**	**0.104**	0.58	0.42	**3.043**	**0.081**
Patients with AH	212	0.39	0.5	0.11			0.64	0.36		
