# Supplementary material for: FOXP2 gene and language impairment in schizophrenia: association and epigenetic studies
Source: BMC Med Genet. 2010 Jul 22;11:114. doi: 10.1186/1471-2350-11-114 (PMC2918571; doi:10.1186/1471-2350-11-114)
Supplement: Additional file 6 — Genotype and allele frequencies of the analyzed SNPs in patients with auditory hallucinations (AH) and patients without AH. a tests in which expected values for more than one class are lower than 5. b tests in which expected values for one class are lower than 2. c tests in which due to lack of some classes, it was used a table 2 × 2 instead a 3 × 2. * it corresponds to corrected p value (Bonferroni correction). [file 1471-2350-11-114-S6.PDF]

SNP		Genotype frequencies			χ^2	P	Allelic frequencies		χ^2	P *
**rs7803667**		**TT**	**TA**	**AA**			**T**	**A**		
Patients with AH	77	0.4	0.51	0.09	**0.398**	**0.839**	0.66	0.34	**0.044**	**0.835**
Patients without AH	215	0.43	0.47	0.1			0.67	0.33		
**rs10447760**		**CC**	**CT**	**TT**			**C**	**T**		
Patients with AH	77	0.44	0.48	0.08	**0.410**	**0.836**	0.68	0.31	**0.342**	**0.559**
Patients without AH	215	0.48	0.45	0.07			0.71	0.29		
**rs6961558**		**GG**	**GA**	**AA**			**G**	**A**		
Patients with AH	76	0.97	0.03	0	**0.201**[c]	**0.738**	0.99	0.01	**0.197**	**0.657**
Patients without AH	215	0.96	0.04	0			0.98	0.02		
**rs923875**		**AA**	**AC**	**CC**			**A**	**C**		
Patients with AH	77	0.27	0.57	0.16	**1.238**	**0.546**	0.56	0.44	**0.558**	**0.455**
Patients without AH	215	0.34	0.51	0.15			0.59	0.41		
**rs1597548**		**CC**	**CG**	**GG**			**C**	**G**		
Patients with AH	77	0.95	0.05	0	**2.927**[c]	**0.062**	0.97	0.03	**2.768**	**0.096**
Patients without AH	215	0.88	0.12	0			0.94	0.06		
**rs10500038**		**CC**	**CT**	**TT**			**G**	**A**		
Patients with AH	64	0.69	0.31	0	**3.458**[a]	**0.196**	0.84	0.16	**1.771**	**0.183**
Patients without AH	200	0.63	0.32	0.05			0.79	0.21		
**rs4730626**		**GG**	**GA**	**AA**			**G**	**A**		
Patients with AH	61	0.67	0.31	0.02	**0.986**[a]	**0.599**	0.83	0.17	**0.019**	**0.892**
Patients without AH	200	0.68	0.28	0.04			0.82	0.18		
**rs1668335**		**GG**	**GA**	**AA**			**G**	**A**		
Patients with AH	62	0.6	0.27	0.13	**4.704**	**0.097**	0.73	0.27	**0.126**	**0.722**
Patients without AH	200	0.51	0.42	0.08			**0.72**	**0.28**		
**rs11771168**		**CC**	**CT**	**TT**			**C**	**T**		
Patients with AH	64	0.59	0.34	0.06	**0.456**[a]	**0.804**	0.77	0.23	**0.416**	**0.519**
Patients without AH	200	0.63	0.32	0.04			**0.79**	**0.21**		
**rs1916977**		**AA**	**AG**	**GG**			**A**	**G**		
Patients with AH	62	0.58	0.34	0.08	**0.371**[a]	**0.895**	0.75	0.25	**0.052**	**0.820**
Patients without AH	200	0.58	0.36	0.06			0.76	0.24		
**rs2396722**		**TT**	**TC**	**CC**			**T**	**C**		
Patients with AH	77	0.39	0.49	0.12	**0.628**	**0.781**	0.64	0.36	**0.03**	**0.862**
Patients without AH	215	0.42	0.44	0.13			0.64	0.36		
**rs2253478**		**GG**	**GA**	**AA**			**G**	**A**		
Patients with AH	64	0.28	0.62	0.09	**6.487**	**0.039**<br>**0.936**	0.59	0.41	**0.641**	**0.423**
Patients without AH	199	0.41	0.44	0.15			0.63	0.37		
**rs2694941**		**TT**	**TA**	**AA**			**T**	**A**		
Patients with AH	62	0.37	0.47	0.16	**0.440**	**0.809**	0.60	0.40	**0.451**	**0.502**
Patients without AH	198	0.33	0.47	0.19			0.57	0.43		
**rs1852469**		**AA**	**AT**	**TT**			**A**	**T**		
Patients with AH	77	0.92	0.08	0	**0.361**[b]	**1.000**	0.96	0.04	**0.076**	**0.783**
Patients without AH	215	0.92	0.08	0.005			0.96	0.04		
**rs10255943**		**GG**	**GA**	**AA**			**G**	**A**		
Patients with AH	62	0.48	0.44	0.08	**0.126**	**0.954**	0.70	0.30	**0.088**	**0.766**
Patients without AH	200	0.47	0.44	0.1			0.69	0.31		
**rs10486026**		**TT**	**TC**	**CC**			**T**	**C**		
Patients with AH	62	0.6	0.34	0.6	**1.068**[a]	**0.636**	0.77	0.23	**0.562**	**0.454**
Patients without AH	200	0.63	0.34	0.04			0.79	0.21		
**rs2396753**		**AA**	**AC**	**CC**			**A**	**C**		
Patients with AH	77	0.35	0.49	0.16	**6.611**	**0.037**<br>**0.888**	0.60	0.40	**6.605**	**0.010**<br>**0.24**
Patients without AH	215	0.21	0.53	0.26			0.48	0.52		
**rs17137124**		**TT**	**TC**	**CC**			**T**	**C**		
Patients with AH	77	0.23	0.47	0.3	**3.512**	**0.169**	0.47	0.53	**3.55**	**0.059**
Patients without AH	215	0.32	0.48	0.2			0.56	0.44		
**rs7799652**		**TT**	**TG**	**GG**			**T**	**G**		
Patients with AH	64	0.33	0.42	0.25	**3.021**	**0.222**	0.54	0.46	**0.837**	**0.360**
Patients without AH	200	0.33	0.51	0.16			0.58	0.42		
**rs1456029**		**AA**	**AG**	**GG**			**A**	**G**		
Patients with AH	64	0.56	0.39	0.05	**1.030**[a]	**0.630**	0.76	0.24	**0.322**	**0.571**
Patients without AH	200	0.55	0.36	0.08			0.73	0.27		
**rs12670585**		CC	CT	TT			C	T		
Patients with AH	62	0.52	0.39	0.1	**0.343**[a]	**0.837**	0.71	0.29	**0.037**	**0.848**
Patients without AH	199	0.51	0.41	0.08			0.72	0.28		
**rs1456031**		**TT**	**TC**	**CC**			**T**	**C**		
Patients with AH	77	0.36	0.35	0.29	**6.611**	**0.037**<br>**0.888**	0.54	0.46	**1.028**	**0.311**
Patients without AH	215	0.34	0.49	0.17			0.59	0.41		
**rs2396765**		**TT**	**TC**	**CC**			**T**	**C**		
Patients with AH	64	0.41	0.42	0.17	**1.754**	**0.435**	0.62	0.38	**0.033**	**0.854**
Patients without AH	198	0.37	0.51	0.12			0.63	0.37		
**rs1456021**		**TT**	**TG**	**GG**			**T**	**G**		
Patients with AH	65	0.42	0.43	0.15	**1.266**	**0.536**	0.63	0.37	**0.016**	**0.901**
Patients without AH	212	0.39	0.5	0.11			0.64	0.36		
